# Successful treatment with tocilizumab for refractory anemia and slowly progressive renal glomerulosclerosis in multicentric Castleman disease: A case report

**DOI:** 10.1097/MD.0000000000028941

**Published:** 2022-02-25

**Authors:** Eri Sugawara, Taiki Sato, Yoshiharu Amasaki, Kazuaki Katsumata

**Affiliations:** a Department of Rheumatology, Tonan Hospital, Sapporo, Japan; b Kuriyama Red Cross Hospital, Kuriyama, Japan.

**Keywords:** anemia, case report, Catsleman disease, chronic kidney disease, tocilizumab

## Abstract

**Rationale:**

Multicentric Castleman disease (MCD) is a rare lymphoproliferative disorder accompanied by systemic symptoms characterized by polyclonal hypergammaglobulinemia and chronic inflammation due to overexpression of interleukin-6. Histological heterogeneity of renal involvement in MCD has been described, although the number of reports is limited. Tocilizumab, a humanized anti-interleukin-6 receptor antibody, has been reported to be effective for MCD.

**Patent concerns:**

A 64-year-old man experienced refractory anemia and slowly progressive renal dysfunction with proteinuria, accompanied by persistent inflammation for 11 years.

**Diagnosis:**

Two renal biopsies were obtained. The first biopsy performed 7 years before admission revealed non-specific interstitial inflammation, whereas the second biopsy demonstrated global sclerosis in most glomeruli and interstitial fibrosis. The patient had multiple lymphadenopathies. Cervical lymph node biopsy histological findings were compatible with plasma cell type Castleman disease. The patient had no evidence of human hepatitis virus-8 infection.

**Intervention:**

The patient was treated with 60 mg/d prednisolone followed by 8 mg/kg intravenous tocilizumab every 2 weeks.

**Outcome:**

His anemia significantly improved, as well as a marked reduction in proteinuria and stabilization of renal function. He did not experience renal function during the 2-years follow-up period.

**Lessons:**

The heterogeneity of the renal manifestations of MCD sometimes makes early diagnosis difficult. We need to interpret the histological findings of the renal biopsy carefully. For advanced-stage renal diseases, tocilizumab might be an effective treatment strategy for MCD.

## 1 Introduction

Castleman disease is a lymphoproliferative disorder with benign hyperplastic lymph nodes characterized by follicular hyperplasia and capillary proliferation with endothelial hyperplasia,^[1]^ which is histologically classified as hyaline-vascular, plasma cell type, or mixed type and clinically classified as localized or systemic (multicentric). Although the etiology of multicentric Castleman disease (MCD) remains unknown, interleukin-6 (IL-6), a proinflammatory cytokine, is thought to play a central role in the pathogenesis of MCD.^[2]^ Recently, tocilizumab, a humanized anti-IL-6 receptor antibody, was reported to be effective against MCD.^[3]^ Renal involvement associated with MCD has been described in a limited number of case reports and case series^[4]^; however, its histopathological findings are heterogeneous, including mesangial proliferative glomerulonephritis, membranoproliferative glomerulonephritis, interstitial nephritis, and amyloidosis.^[5]^ Herein, we report a case of MCD with refractory anemia, slowly progressive renal dysfunction, and proteinuria accompanied by persistent inflammation in a patient who was treated with tocilizumab.

## 2 Case presentation

**Figure 1:**
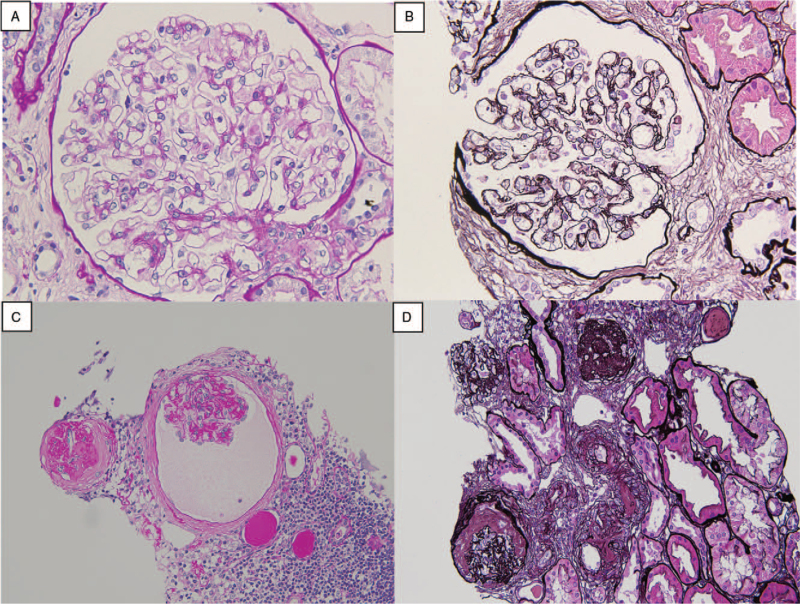
Renal biopsy in 2012 revealed arterial sclerosis and mild interstitial infiltration of inflammatory cells by periodic acid-Schiff (PAS) staining (A) and periodic acid-methenamine-silver (PAM) staining (B). Renal biopsy in 2019 demonstrated global sclerosis of 9 of 12 glomeruli by PAS staining (C) and intestinal fibrosis by PAM staining (D). There was no evidence of mesangial proliferation, plasma cell infiltration and amyloid deposits.

**Figure 2:**
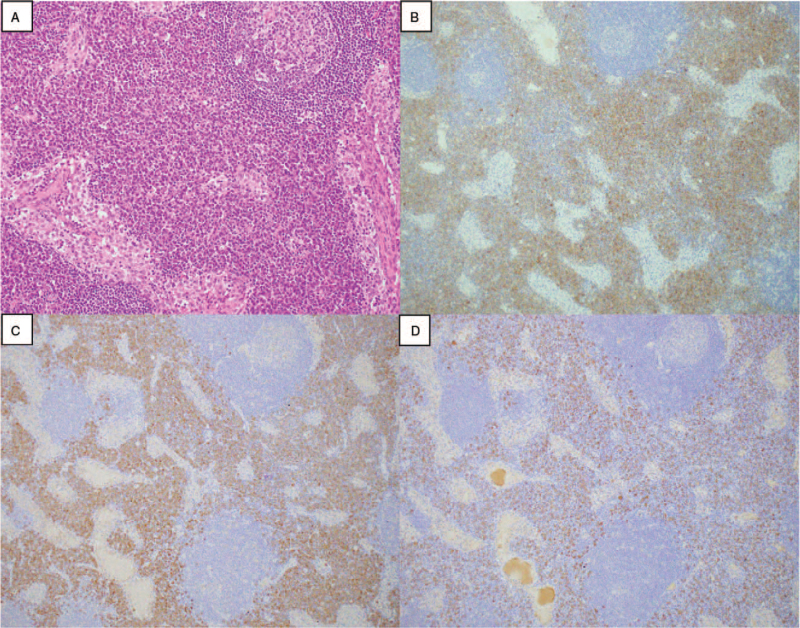
Histology of the cervical lymph node, showing follicular hyperplasia and diffuse plasma cell proliferation by hematoxylin-eosin staining (A) and CD138 staining (B). Immunohistochemical staining for IgG (C) and IgG4 (D) revealed and the IgG4 + /IgG+ plasma cell ratio was 30%.

**Figure 3:**
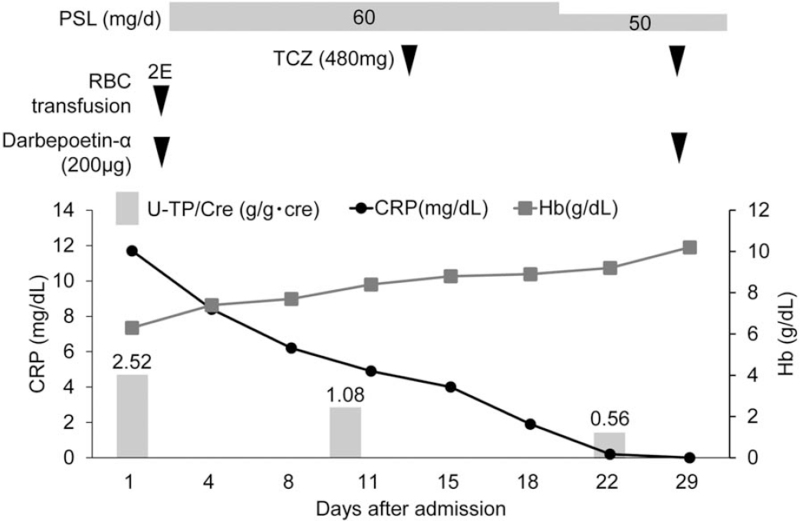
Clinical course of the patient. CRP = C-reactive protein, Hb = hemoglobin, PSL = prednisolone, RBC = red blood cell, TCZ = tocilizumab, U-TP = urinary protein-to-creatinine ratio.

A 64-year-old man had progressive anemia, proteinuria, polyclonal hypergammaglobulinemia, and elevated C-reactive protein (CRP) since 2008. Renal biopsy in 2012 demonstrated arterial sclerosis and mild interstitial infiltration of inflammatory cells (Fig. 1A and B). Bone marrow trephine biopsy revealed no evidence of hematologic malignancy. Despite monthly cutaneous injection of 100 μg of darbepoetin alpha, he experienced persistent anemia and required occasional RBC infusion. Due to refractory anemia and exertional dyspnea, he admitted to our hospital in October 2019, he had marked anemia (Hb 6.7 g/dL), elevated CRP (13.2 mg/dL), excess IL-6 (60.6 pg/mL), elevated total protein (11.3 g/dL), hypoalbuminemia (2.3 g/dL), renal dysfunction (blood urea nitrogen 37 mg/dL, serum creatinine 2.3 mg/dL, creatinine clearance 35 mL/min) elevated ferritin (477 ng/mL) as well as hypergammaglobulinemia (Immunoglobulin [Ig]G 6585 mg/dL, IgA 809 mg/dL, IgM 195 mg/dL, IgG4 1240 mg/dL) without monoclonal peak on immunoelectrophoresis in either serum or urine. Anti-nuclear antibodies, anti-neutrophil cytoplasmic antibodies, and anti-glomerular basement membrane antibodies were not detected. Hepatitis B surface antigen, hepatitis C antibody, human hepatitis virus-8 DNA, and cryoglobulins were not detected. Urinalysis showed microhematuria without any casts, non-nephrotic range proteinuria (2.5 g/d), and a urinary protein-to-creatinine ratio of 0.9 g/gCr. Renal biopsy revealed global sclerosis in 9 of 12 glomeruli and interstitial fibrosis without evidence of mesangial proliferation, plasma cell infiltration, or amyloid deposits (Fig. 1C and D). Immunofluorescence analysis showed no significant staining of glomeruli. Computed tomography from the neck to the pelvis revealed multiple lymphadenopathies and splenomegaly. A cervical lymph node biopsy revealed follicular hyperplasia and diffuse plasma cell proliferation (Fig. 2A and B), which was compatible with plasma cell type Castleman disease. IgG4 positive plasma cells were observed, and the IgG4 + /IgG+ plasma cell ratio was 30% (Fig. 2C and D), which did not meet the comprehensive diagnostic criteria for IgG4-related disease (IgG4-RD).^[6]^ The patient was diagnosed with MCD and treated with 60 mg/d of prednisolone (PSL) followed by 8 mg/kg of intravenous tocilizumab every 2 weeks. His anemia, inflammation, and polyclonal gammopathy improved, and his renal function stabilized (Fig. 3). His exertional dyspnea also improved. The PSL dose was gradually tapered to a low maintenance dose. The patient experienced no adverse events. At the last available follow-up in October 2021, the serum creatine level was 2.4 mg/dL and urinary protein-to-creatinine ratio was 1.0 g/gCr.

## 3 Discussion

Herein, we report a case of MCD with chronic renal failure and refractory anemia that was successfully treated with tocilizumab. MCD is a benign lymphoproliferative disease characterized by lymphadenopathy and polyclonal hypergammaglobulinemia.^[1]^ Although the etiology of MCD remains unknown, IL-6, a pro-inflammatory cytokine, is thought to play a central role in the pathogenesis of MCD.^[2]^

Renal involvement associated with MCD has been described in a limited number of case reports and series. Based on these studies, renal histopathological features of MCD are heterogeneous, including mesangial proliferative glomerulonephritis,^[7]^ membranoproliferative glomerulonephritis,^[8]^ interstitial nephritis,^[9]^ crescentic glomerulonephritis,^[10]^ and amyloidosis.^[11]^ In addition to its other manifestations, IL-6 plays a key role in the pathogenesis of renal MCD. IL-6 regulates mesangial cell growth in an autocrine manner.^[12]^ Furthermore, dysregulated expression of IL-6 causes excessive production of vascular endothelial growth factor (VEGF) in an IL-6-dependent manner.^[13]^ Although the precise role of VEGF in the pathogenesis of renal manifestations of MCD remains to be elucidated, overexpressed VEGF might cause abnormal proliferation of endothelial cells, leading to glomerular changes.^[8]^

MCD is sometimes accompanied by the elevation of serum IgG4 and infiltration of IgG4+ plasma cells in the affected organs, which could fulfil the diagnostic criteria for IgG4-RD,^[14]^ making it difficult to differentiate MCD from IgG4-RD. However, clinical features, including distribution of organ involvement, atopic history, and levels of IgA and CRP, are reported to be helpful in differentiating between diseases.^[15]^

To the best of our knowledge, there are no reports on the long-term renal prognosis of MCD. In the current case, despite the low-grade interstitial inflammation in renal biopsy in 2012, which seemed to be a non-specific finding, a second renal biopsy in 2019 revealed advanced glomerulosclerosis and interstitial fibrosis, suggesting the difficulties of early diagnosis of renal manifestations of MCD. We should have interpreted non-specific renal inflammation more carefully considering the heterogeneity of renal involvement in MCD. Tocilizumab has been reported to be effective for secondary amyloid A amyloidosis^[16]^ and anemia in end-stage renal disease with MCD.^[17]^ In the current case, tocilizumab improved his refractory anemia and stabilized renal function, suggesting a reno-protective effect. Therefore, tocilizumab should be considered in patients with MCD, even in those with advanced-stage renal diseases. Further studies are needed to confirm treatment strategies for MCD.

## Acknowledgment

The authors thank the patient for his collaboration. They thank Hiroko Takeda and Yayoi Ogawa for pathologic diagnosis, Naoki Hyakushima for cervical lymph node biopsy, and Rintaro Machino for renal biopsy and helpful discussions.

## Author contributions

**Writing – original draft:** Eri Sugawara.

**Writing – review & editing:** Eri Sugawara, Taiki Sato, Yoshiharu Amasaki, Kazuaki Katsumata.
